# Current status and prospects of automatic sleep stages scoring: Review

**DOI:** 10.1007/s13534-023-00299-3

**Published:** 2023-07-10

**Authors:** Maksym Gaiduk, Ángel Serrano Alarcón, Ralf Seepold, Natividad Martínez Madrid

**Affiliations:** 1 HTWG Konstanz – University of Applied Sciences, Alfred-Wachtel-Str.8, 78462 Konstanz, Germany; 2 Reutlingen University, Alteburgstraße 150, 72762 Reutlingen, Germany

**Keywords:** Automatic sleep assessment, Sleep scoring, Physiological signals, Sleep stages

## Abstract

The scoring of sleep stages is one of the essential tasks in sleep analysis. Since a manual procedure requires considerable human and financial resources, and incorporates some subjectivity, an automated approach could result in several advantages. There have been many developments in this area, and in order to provide a comprehensive overview, it is essential to review relevant recent works and summarise the characteristics of the approaches, which is the main aim of this article. To achieve it, we examined articles published between 2018 and 2022 that dealt with the automated scoring of sleep stages. In the final selection for in-depth analysis, 125 articles were included after reviewing a total of 515 publications. The results revealed that automatic scoring demonstrates good quality (with Cohen's kappa up to over 0.80 and accuracy up to over 90%) in analysing EEG/EEG + EOG + EMG signals. At the same time, it should be noted that there has been no breakthrough in the quality of results using these signals in recent years. Systems involving other signals that could potentially be acquired more conveniently for the user (e.g. respiratory, cardiac or movement signals) remain more challenging in the implementation with a high level of reliability but have considerable innovation capability. In general, automatic sleep stage scoring has excellent potential to assist medical professionals while providing an objective assessment.

## Introduction

Sleep plays a vital role in human life. Its influence on physical and psychological health and human well-being is enormous, which has already been demonstrated and underlined in numerous publications [1–4]. Accordingly, it is crucial to be able to analyse sleep to have the possibility to intervene in time in case of disorders, to eliminate them if possible, or at least to reduce their negative influence on health.

As early as the 1950s, William Dement observed that the signal of the electroencephalogram (EEG), the frequency of eye movements and the frequency of body movements were subject to regular cyclical fluctuations during the night [5]. Further research into this phenomenon led over time to the recognition of typical sleep patterns and ultimately to the creation of the concept of sleep stages.

In 1968, the first standardised categorisation of sleep into sleep stages was conducted using the method of Rechtschaffen and Kales (R&K) [6]. It divided sleep into 30-s intervals—"epochs"—and assigned one of the following sleep stages to each epoch:

- Stage W—Wakefulness/Wakefulness
- Stage 1 (S1)
- Stage 2 (S2)
- Stage 3 (S3)
- Stage 4 (S4)
- Stage REM—Rapid eye movement

Furthermore, some epochs could be labelled "movement time" if movement prevents accurate identification of sleep stages.

Over time, as new evidence about sleep emerged, there was a need to establish a new guide to terminology, recording methodology and scoring rules for sleep. This was done in 2007 with the release of a new guideline by the American Academy of Sleep Medicine (AASM) [7], which has been continuously updated since its release. The most recent version is 2.6, published in 2020 [8]. It proposed, among other things, a new classification of sleep stages:

- Stage W—wakefulness
- Stage N1/NREM1 (formerly S1)
- Stage N2/NREM2 (formerly S2)
- Stage N3/NREM3 (formerly S3 + S4)
- Stage R—Rapid eye movement (REM)

In fact, inter-standard comparison of sleep stage scoring has been the focus of several scientific publications, among them [9, 10].

In sleep analysis practice, sleep stages N1-N3 are, in some cases, combined into one NREM stage. Another subdivision found in scientific papers is Wake/Light Sleep (N1 + N2)/Deep Sleep (N3)/REM sleep (R). Table 1 summarises these sleep stage classification manners.

**Table 1:** Sleep stages classification

Number of sleep stages	Sleep stages				
5	W	N1	N2	N3	R
4	Wake	Light		Deep	R
3	Wake	NREM			R
2	Wake	Sleep			

The most widely utilised method for assessing sleep behaviour, which has been in use for many years, is polysomnography (PSG) [11]. This approach involves measuring several signals, which are then evaluated:

- Electroencephalography (EEG) records brain activity.
- Electrocardiography (ECG) is a method that reflects the electrical activity of the heart over time.
- Electromyography (EMG) is used to record muscle activity.
- Electrooculography (EOG), on the other hand, uses electrodes to detect and measure the potential between the human eye's back and front to record eye movements.
- The oxygen saturation of the blood is measured with pulse oximetry.
- Often, both respiratory flow and respiratory effort are measured.
- Moreover, other signals can be recorded, such as the person's position or a video recording for a detailed analysis.

The recorded signals are then stored and finally analysed manually by trained sleep experts, scoring the stages of sleep. Mainly EEG, EOG, and EMG signals provide the required input.

This manual evaluation of the recordings enables accurate scoring of the sleep stages. However, it also entails a high expenditure of time and financial resources to complete this task [12]. It should also be noted that despite following the established scoring guidelines, manual analysis introduces a certain level of subjectivity, also known as interscorer/interrater reliability/variability, which has been described in several scientific publications [13–15]. Thus, the results of the evaluation of the same sleep recording by different experts may vary. As the meta-analysis of interrater reliability conducted in [16] has demonstrated, Cohen's kappa for manual, overall sleep scoring reaches the value of 0.76.

To summarise the above situation, automatic sleep scoring could result in several benefits. Among other things, it could reduce the financial and human resources needed, thereby supporting the work of sleep physicians and making them available to provide treatment to a larger number of individuals requiring it. A high number of scientific works in the field of automatic sleep stage detection indicate that new developments are constantly being made in this area, and only a thorough analysis can provide a comprehensive overview of the status quo.

The conducted analysis of current research has indicated that the subject of a state-of-the-art review in automatic sleep stage identification has been addressed several times, highlighting the importance of this topic. However, studies in recent years have focused on specific aspects of the problem. For example, in [17], only the algorithms that work with the EEG signal were selected. In [18], consumer sleep technologies (CSTs) were investigated to analyse sleep in combination with artificial intelligence. [19] examined a more extended period of 19 years but with a relatively small number of selected articles (55 in total) for this larger time frame. EEG signals in combination with deep learning algorithms applied directly to raw signals or spectrograms, were the subject of investigation in [20], resulting in a more refined but smaller sample of 14 studies. Deep learning techniques for sleep phase detection were investigated in [21], with 36 studies selected from the period between 2010 and 2020. The number of studies reviewed in [22] is also relatively small, below 30, which is in part caused by the research question in which EEG signal-based algorithms were investigated. Also, if one looks further into the past, it can be seen that the matter of automatic detection of sleep phases was addressed considerably earlier, and was, for example, already investigated in a review article in 2012 [23]. In general, the principles of automatic sleep scoring were even reported in 2000 [24], indicating the topic's importance and demonstrating a long history of research in this scientific area.

Due to the major importance of the topic, knowing the enormous number of recent developments in the field of automatic sleep scoring and considering the gaps in existing review articles in recent years, the decision was made to conduct a detailed investigation of state-of-the-art and to report the results in a review article. The aim is to prepare a comprehensive overview of the state-of-the-art in the field of automatic sleep stage identification and provide researchers with a consolidated summary of the current developments, which should increase the efficiency of their scientific research.

Several approaches to literature reviews exist. One of the classifications proposes the following groups: systematic, semi-systematic (or narrative) and integrative, according to [25]. In our case, it seems appropriate to use the semi-systematic approach, as it facilitates more flexibility and is commonly used for overview publications. In addition to aiming to provide an overview of a topic, a semi-systematic review often examines how research in a particular area has developed over time [25]. This type of review can be a highly effective way to cover more areas and broader topics than a systematic review can address, especially when multiple subject domains (such as computer science and medicine) are in the field of interest [26].

Three main research questions are being addressed in our article:

1. Which level of quality is achieved at the current state of development of automatic sleep scoring approaches?
2. How has the focus on particular signal analysis methods evolved over time?
3. How has the choice of signals used for analysis varied over time?

## Materials and methods

### Eligibility criteria

A list of inclusion and exclusion criteria was established based on the analysis of the current state of the art and considering the defined research questions.

**Inclusion criteria**

- Automatic approach for sleep scoring is described in a manuscript
- Peer-reviewed journals
- Publications considering adults (> 18) as a group of interest
- English language
- Manuscripts published between 2018 and 2022
- Ground truth labels came from AASM rules

**Exclusion criteria**

- Article size of fewer than four pages
- Test dataset is smaller than 20 overnight recordings
- Animals being a subject of investigation
- Only Wake/Sleep identification without more detailed scoring
- Review article/Editorial/Book chapters (however, these arts of publications are being checked for eligible articles)
- Epochs of longer than 30 s are being analysed
- Missing significant information on some of the points from the exclusion criteria list

When a research group has published several consecutive articles that represent a further development of particular methods, only the most recent article has been included in this review. If the reported approaches represent significantly different procedures or use diverse input signals, all of them are included in the selection.

A more detailed explanation of the inclusion criterion "Automatic approach for sleep scoring is described in a manuscript" appears to be reasonable. As one of the important aspects for the developed sleep stage identification system is its applicability in practice and usability, only those publications were included in the review that allow fully automatic scoring, i.e. the systems that require manual intervention in use, such as partial manual scoring, were not considered in this work.

In 2007, the AASM guidelines [7] replaced the Rechtschaffen & Kales rules [6] as the new standard for sleep assessment. Therefore, in the selection of articles, the publications were included in which the sleep phase detection of the reference measurement is done according to AASM guidelines, as only the manuscripts since 2018 were considered. However, in order to be able to evaluate the methods that were using the older databases already available, the decision was made also to consider the articles that were using the existing recordings according to R&K rules, but adapting them to the sleep stages defined in AASM.

### Information collection strategy

After analysing the requirements for articles to be included in the review and considering the interdisciplinary nature of the topic, the following selection of databases was made to ensure the comprehensive collection of information:

- IEEE Xplore
- PubMed
- Scopus
- Web of Science

The following query (with necessary adaptions for specific databases) was determined to search for articles that match the defined criteria:

(((automatic OR automated OR automatically) AND ("sleep stage" OR "sleep stages" OR "sleep phase" OR "sleep phases")) AND (scoring OR classification OR determination OR identification OR recognition OR detection)). The search using this defined query took place on the articles' titles, abstracts and keywords in databases. In addition, a restriction of years (2018–2022) was used, either directly in the query, if the database provided this option, or through the later application of an appropriate filter.

### Data refining procedure

The next step after applying the designed query in the selected databases was to refine the collected data. This involved several steps. First, duplicates were removed, and all non-journal articles were excluded from the list of articles. This was followed by three phases of initial screening: title, abstract and diagonal reading. During this process, the eligibility of the manuscripts was checked according to the inclusion/exclusion criteria, and if they did not meet them, they were eliminated. At last, the final screening was applied, where the full reading of the papers was performed. In this step, the last articles that did not meet the established criteria were removed to generate the final list of manuscripts for further analysis.

### Synthesis methods

A set of Python packages (Pandas, NumPy, Seaborn, Matplotlib and SciPy) were used for the statistical analysis carried out in this scientific work. Statistical data visualisations were presented to aid the understanding of the results. Where possible, statistics were proposed to explain relevant information gathered during the review of the articles.

## Results

The conducted in-depth analysis of the current research outcomes over the last five years has led us to create a comprehensive overview, which is presented in the following. It is important to point out that not all descriptions of the implementation and evaluation approaches provide sufficient information to estimate the results' quality and correctness. Nevertheless, this review has included these articles according to inclusion/exclusion criteria.

### Study selection

**Fig. 1:**
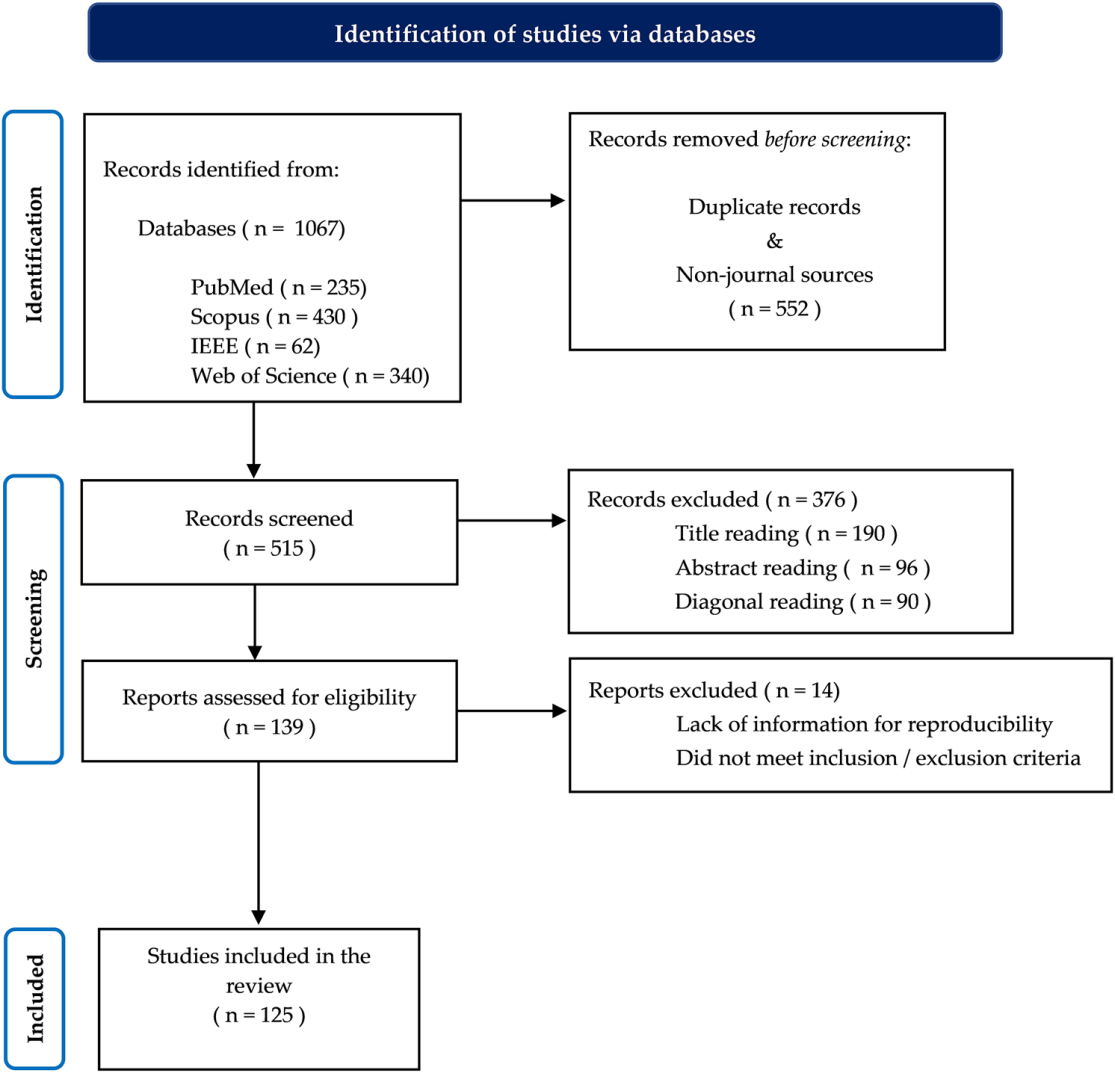
Flow chart for the selection of the entire set of the included publications

The article selection process carried out throughout this review of scientific papers dealing with the classification of sleep stages is shown in Fig. 1. The number of publications included and excluded during each review stage is also shown.

### Which level of quality is achieved at the current state of development of automatic sleep scoring approaches?

A summary table was prepared to provide a comprehensive overview of the available approaches for automatic sleep stage scoring, including all articles selected according to inclusion criteria and after refining the set according to exclusion criteria. The results can be seen in Table 2, attached to this manuscript. Due to its comprehensive content, inserting it directly into the text would significantly decrease the manuscript's readability, which should be avoided.

The best accuracies reported in the reviewed manuscripts achieve over 90% when analysing EEG signal, being an excellent result. As reported in several research papers, the best conducted Cohen’s kappa values are over 0.80, and F1 values achieve up to 85–90%.

The differences in performance of some algorithms can vary significantly depending on the composition of the test group/dataset. In particular, if the training was done on healthy subjects only, testing on a group with sleep disorders (e.g., sleep apnoea) would be associated with a high probability of a reduction in sleep stage detection accuracy, as indicated in, e.g. [27]. Therefore, the provided table includes information on a targeted population of the developed algorithms.

In the column “Algorithm/Method” in the table, only the primary used approach for sleep scoring is provided. Feature extraction/filtering procedures, being another relevant characteristic of the method, are not described due to the large variability and individuality of utilised approaches which complicates their classification and would excessively enlarge the table, decreasing its clarity.

### How has the focus on particular signal analysis methods evolved over time?

**Fig. 2:**
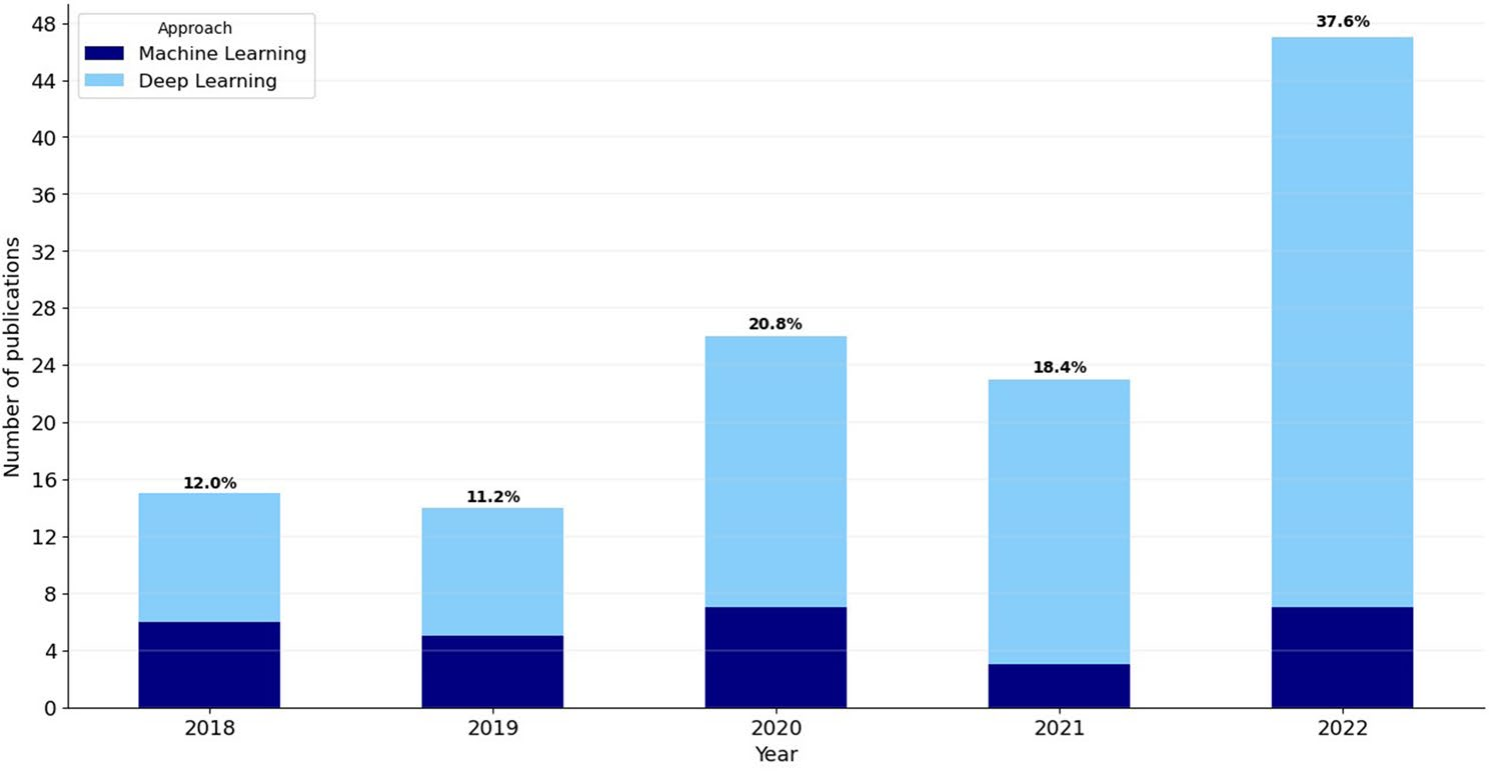
Bar plots with the number of publications that met the inclusion/exclusion criteria. The terms “Machine Learning” (with Deep Learning excluded) and “Deep Learning” refer to the approach followed for sleep stage classification

Trends in research are changing over time, among other things, as new knowledge is gained and new priorities may be set. This raises the question of how the choice of methods for sleep stage estimation has evolved over the last few years. Figure 2 depicts the list of publications that met the inclusion/exclusion criteria and were selected for statistical analysis separately for the years 2018–2022, as well as the breakdown by primary approach for every single year. The highest number of selected articles was published in 2022 (37.6% of all publications), and the lowest number of selected papers was in 2018 and 2019. It can also be seen in Fig. 2 that the use of deep learning techniques is much higher than other machine learning approaches in any year from 2018 to 2022.

### How has the choice of signals used for analysis varied over time?

**Fig. 3:**
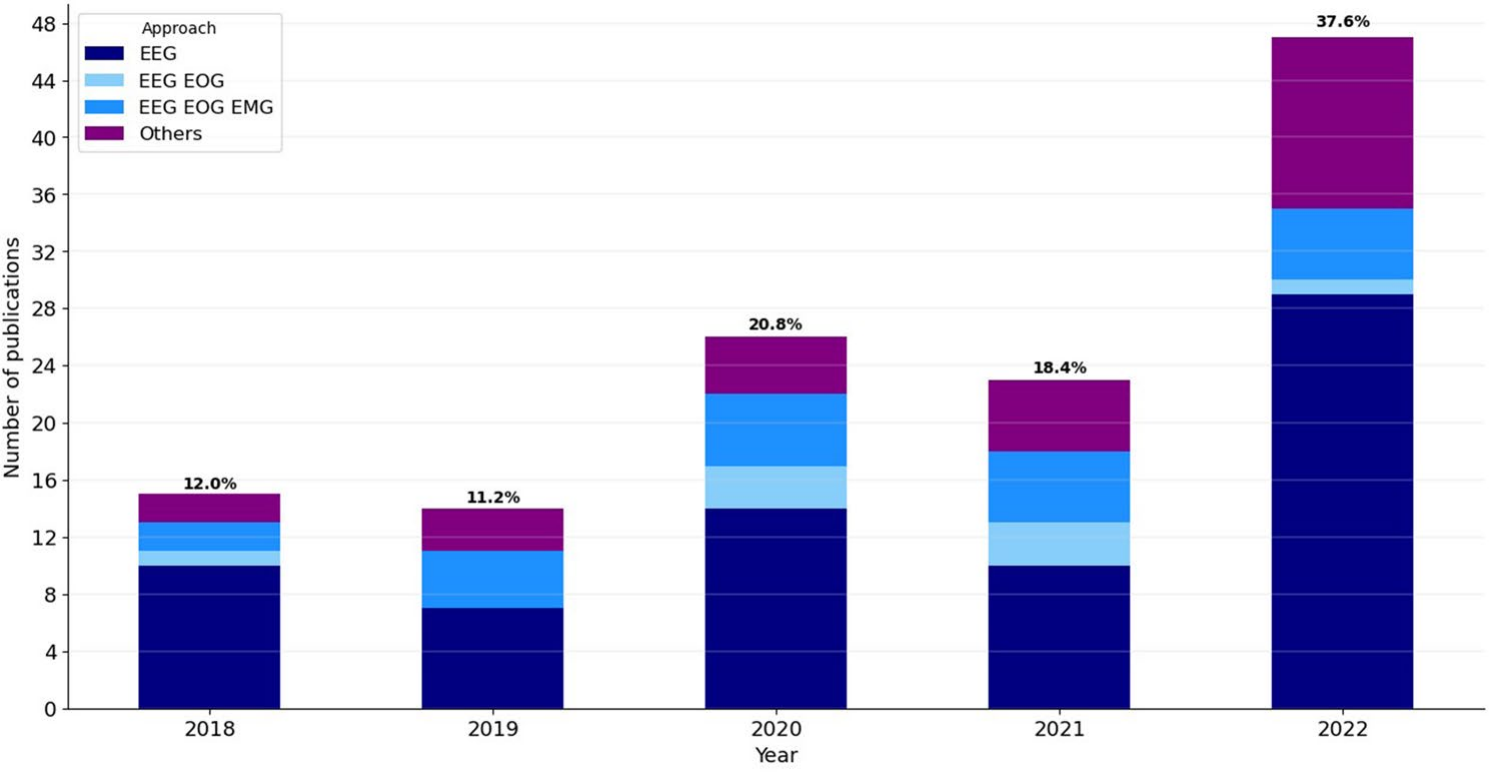
Bar plots with the number of publications per year depending on the signal used. Four main groups of signals analysed for sleep stage classification are represented: "EEG", "EEG + EOG", "EEG + EOG + EMG", and "Others"

The selection of signals for analysis with the subsequent scoring of sleep stages and their variation over time also provides interest for the research. Therefore, we have graphically presented the selection of signals by year in Fig. 3 to facilitate an overview and to illustrate possible trends.

**Fig. 4:**
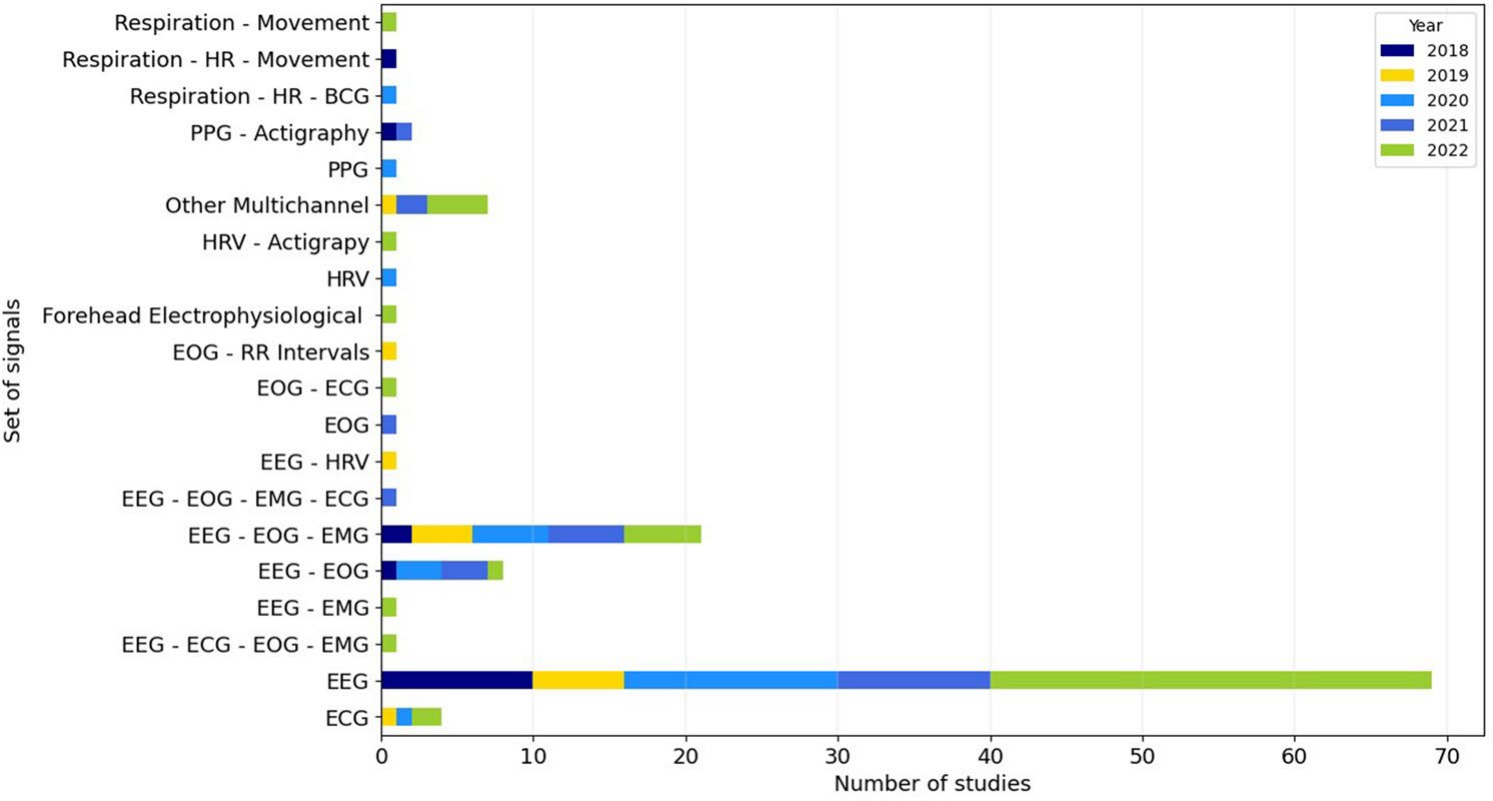
Set of signals used by the reviewed scientific works for classifying sleep stages from 2018 to 2022

With the aim of providing more insight into the selection of signals used to score sleep stages in the articles included in the review, the detailed representation per input signal was generated and can be seen in Fig. 4.

As shown in Fig. 4, the EEG signal is the most widely used as opposed to other signal sets, with 69 scientific publications using standalone EEG and being a part of numerous other combinations with other signals.

## Discussion

The findings of the conducted review represent, to the best of our knowledge, the most comprehensive research overview in the field of automatic sleep stage scoring in recent years. This allows a thorough analysis of current developments in this domain and can serve as a basis for further research.

Most of the information for the analysis can be found in Table 2. For example, it can be seen that the majority of publications have considered the detection of five sleep stages, which corresponds to the AASM standard. Only occasionally did the methods target fewer sleep stages.

The most common method of validation, as indicated in Table 2, is cross-validation. More specifically, tenfold and 20-fold cross-validation are the most commonly used techniques in the reviewed articles. In addition, other x-fold cross-validation and leave-one-subject-out approaches can be found in publications. Direct strict separation of training and validation/test datasets is also reported in several articles. It is important to note that using different epochs of the same recording for both training and evaluation would affect the algorithm's performance in terms of increasing accuracy. However, using epochs from the same recording for both training and evaluation does not allow us to assess whether the algorithm used would perform similarly if the subjects for the training and test datasets were strictly separated. Therefore, we have tried to extract this relevant information from the reviewed articles. Unfortunately, far from all articles provided this important detail. For those articles where this information was provided, or where the method of validation (e.g. leave-one-subject-out) allowed us to obtain the required data, we can say that in the majority of cases there was a strict separation of subjects into training and test datasets, and therefore the epochs of the same recording were mostly not used for both training and evaluation.

We can highlight some important points by looking at the quality parameters reported in the research papers analysed. By far, the most widely used signal for sleep stage scoring is the EEG, and the combination of EEG, EOG and EMG is the second most frequently used, as can be recognised from Table 2 and Fig. 3. Together, these two sets account for more than two-thirds of all publications. They also yield the best results in scoring—Cohen's kappa up to over 0.80 (substantial to almost perfect according to [28]) and accuracy up to over 90%, which is a very good performance, especially considering that even when evaluating the same recording by different scorers, a kappa of 0.71–0.81 is obtained, as studied in [16]. In general, the use of automated sleep phase detection methods could address the problem of interrater reliability when the same approach is used for analysis in different sleep laboratories (or even in one sleep laboratory instead of multiple experts). This could help to free up clinicians' time for other clinical tasks.

At the same time, it should be noted that there has been no breakthrough in the quality of the results when analysing these signals (EEG, EOG, EMG) over the last five years in terms of performance. This can be explained, among other things, by the high scores already achieved, as mentioned above. If one is looking for research topics with a high innovation potential, other alternatives should perhaps be considered. An example of this could be systems that work with other signals. These could be, for example, cardiac, respiratory and movement signals, as they have the potential to be recorded with more comfort for the user and possibly without contact [29, 30]. The number of works using these alternative signals for sleep scoring is significantly lower, according to the research conducted, and there is noticeable room for improvement. Nevertheless, looking at Fig. 3, we can see that the number of publications using other than classical signal sets as input to the algorithm has increased over the years.

Another important issue in the development of sleep scoring systems is usability and suitability for practical use. Unfortunately, these aspects are not always explicitly considered in publications, although they are of great importance for the transfer of research into practical implementation. For example, it may be advantageous in terms of usability if the algorithms work fully automatically, i.e. do not require manual pre-classification or pre-processing. Another point that could improve the usability of the systems in a practical application is the determination of the measurement uncertainty during sleep stage detection. This would make it possible to see where the results of the automatic evaluation might need to be re-examined because the classification is not unambiguous, which has been recognised by the software, also known as confidence estimation or prediction certainities. Some work has already been done towards this functionality and usability improvement, e.g. [31–34]. In summary, further research aimed at improving the usability of systems in real-world environments can only be welcomed, as the potential is not yet exhausted, as this review has shown.

The explainability/interpretability of algorithms and their results is also a topic with great potential for further research, as it could at least partially help to solve the "black box" problem, especially in deep learning applications. This issue has already been addressed in some of the reviewed articles [32, 35–38], but there is still much room for further investigation to overcome the current challenges. In general, due to the fact that there is an impressive number of articles on sleep scoring, but at the same time a significantly smaller number of practical applications, the question of usability and practicality seems to be one of the relevant ones and should be further investigated.

Another area of research we observed in some of the manuscripts was the use of algorithms that adapt to the signal or recalibrate the features used [39], or incorporate the probabilities of transitions between sleep stages [40–42]. The inclusion of these additional steps in the algorithms to analyse the signal in an individualised way, while taking into account the whole sleep structure, may also lead to more accurate and higher quality sleep stage detection, and we encourage further research in this direction.

It is pretty common that more than one dataset is used for evaluation in the published articles, as can be seen in Table 2. In this case, however, there are often only two datasets used, which are additionally not always large. Though, the essential point to consider is that the algorithms developed should be evaluated on several, preferable large datasets to ensure a high-quality general scoring that is not only dataset specific, as there may be peculiarities in the signals (due to different equipment, pre-processing, etc.) in other series of recordings. This has been addressed in a number of the papers included in the selection but not in all cases. In order to provide a reliable solution that can be applied to a wide range of situations, it is crucial to consider the universality mentioned above and evaluate with multiple data sets from diverse sleep laboratories and recorded with different devices.

An analysis of Fig. 2 reveals that there is a tendency for the number of developments in the area of sleep stage classification to increase steadily, although there are minor fluctuations in this trend from year to year. Proportionally, deep learning methods stand out from other machine learning techniques in this topic area from year to year, taking an increasingly larger share. This may be partly caused by a general increase in the popularity of deep learning methods in research and partly by progress in developing new, more effective techniques that may produce higher-quality results.

While Fig. 3 gives a rather general picture regarding the signals used in literature, Fig. 4 provides more detail. Here it can be seen that, in addition to the two most frequently used signal sets already mentioned (EEG and EEG + EOG + EMG), a third combination occurs significantly more frequently than other signals—EEG + EOG, although significantly less frequently than the first two. In general, it can be said that a combination with the EEG signal occurs more often than possible combinations with other signals in the research. This can be explained by the fact that the EEG traditionally plays a key role in the detection of sleep stages and also provides crucial information. Apart from these three signals, only the ECG is used significantly more often than the remaining signals. Besides that, other signals were only sporadically included in the approaches reported in the reviewed articles. Another interesting finding from the information presented in Table 2 and Fig. 4 is that the majority of the required signals are recorded with electrodes (EEG, EOG, EMG, ECG), which requires direct contact with the body and is not necessarily the most comfortable for users. This shows that methods that allow non-obtrusive measurement of physiological signals are not yet widely used in sleep stage assessment.

Another important point is that while automated analysis can free up physicians' time for other clinical tasks, even with fully automated sleep evaluation there is still a need for the clinician to review the signals to avoid missing potentially important points. It is known that some specific pathologies can only be detected by looking for specific patterns in the signals that may currently be missed by automated scoring systems.

## Conclusions

Analysing the review findings, in the last years, no breakthrough progress in the quality of sleep scoring approaches analysing EEG, EOG and EMG signals could be observed in terms of perfomance, although the most advanced of the current studies demonstrate a good level of quality in the detection of sleep stages. Nonetheless, a number of topics still remain with some gaps and have excellent innovation potential. These include, among others, the application of algorithms to categorise sleep stages that utilise less traditional signals, such as, for instance, breathing, body movement or heart signals. These signals could possibly be recorded in a contactless and more accessible way than the classical PSG approach and with more comfort for users/patients. Therefore, if an acceptable level of measurement quality could be achieved, it could simplify the measurement process and promote the widespread use of sleep scoring systems. This, in turn, would favour the detection of sleep disorders at an early stage.

Another critical issue that still needs to be further addressed is the matter of interoperability of the developed algorithms with different datasets in order to have the capability to apply the designed sleep stage identification systems universally and not only to one specific dataset. This point was addressed in several of reviewed articles (e.g. [43, 44]), where a significant number of datasets was used for the evaluation. Nevertheless, this topic requires further investigation and assessment.

The issues of explainability, usability, practicality and the use of adaptive algorithms are other areas of research that have been addressed in recent years, but are still not sufficiently explored and have significant potential.

In general, automatic sleep scoring has the potential to create an objective approach devoid of some level of subjectivity and, consequently, variance in scoring, present by manual scorers, considered in the literature as interrater reliability. This point was addressed in [45]. The analysis of current research in the area of sleep scoring presented in our article leads us to the conclusion that automated scoring of sleep stages could become a powerful tool supporting physicians in their work and helping to reduce sleep scoring ambiguity by decreasing the level of subjectivity in the analysis process. It is also noteworthy that the use of automated sleep scoring systems could lead to a saving of resources (both human and financial) that could be allocated to provide a more comprehensive medical service to the general public by medical professionals or to treat a larger number of patients with increased time capacity.

## Appendix 1

See Table 2.

**Table 2:** Articles included in the review according to inclusion/exclusion criteria with detailed description

Author	Year	Signals	Algorithm/Method	Database	Nº of subjects	Population characteristics	Evaluation	Sleep Stages	Accuracy	F1	Kappa
Abdollahpour et al. [46]	2020	Multichannel	Transfer Learning Convolutional Neural Network Data Fusion (TLCNN-DF)	Sleep-EDF Expanded Sleep-EDF	8 subjects 20 subjects	18 Healthy subjects 10 effects of temazepam- insomnio	fivefold CV	5 stages	93.58%		0.89
Abdollahpour et al. [47]	2022	EEG (1 channel)	2 × CNN	Sleep EDF Sleep EDFx DREAMS	4 subjects 20 subjects 20 subjects	4 Healthy subjects 20 effects of temazepam—insomnio 20 Healthy subjects	80% Training 20% Test	5 stages	93.48% 93.14% 83.55%		0.89 0.89 0.77
Alickovicet al. [48]	2018	EEG	Denoising with Multiscale principal component analysis (MSPCA) Feature extraction by DWT, Rotational support vector machine (RotSVM)	Sleep -EDF	20 subjects (30 PSG) 10 subject × 2 PSG	20 Healthy subjects 10 effects of temazepam—insomnio	Grand-Subject.Specific Approach (GSSA) 10-Fold CV	5 stages	91.10%		0.8
Alvarez-Estevez Diego et al. [49]	2021	EEG (2 channels) EOG EMG	CNN LSTM	HMC Dublin SHHS Telemetry DREAMS ISRUC	154 subjects 25 PSG 100 PSG 44 PSG 20 PSG 100 PSG	Mixed (healthy and SDB) subjects	80% Training 20% Test	5 stages			0.79 0.70 0.82 0.80 0.83 0.78
An et al. [50]	2022	EEGs	Amplitude-Time Dual-View signal Representation 1D-CNN Attention-based Feature Fusion	Sleep-EDF Expanded Sleep-EDF	8 subjects 20 subjects	20 Healthy subjects 10 effects of temazepam—insomnio	tenfold CV	6 stages	89.94% 92.07%	84.61% 83.44%	
Arslan et al. [51]	2022	Multichannel	DNN	Own data	50 subjects	50 Healthy subjects	50-fold CV	5 stages	91.6%	90.7%	
Baek et al. [35]	2022	EEG (1 channel)	NA-BEMD (Noise assisted bivariate emprirical mode decomposition) 1D-CNN Bi-LSTM	Sleep-EDF-2013 Sleep-EDF-2018 WSC	20 subjects 78 subjects 116 subjects	214 Healthy subjects	20-fold CV	5 stages	86.22%	80.79%	0.81
Barua et al. [52]	2022	EEG	L-tetrolet TSRFINCA (ReliefF and INCA)	CAP	108 subjects	16 Healthy subjects 92 Pathological (insomnia, SDB, PLM)	tenfold CV	6 stages	91.05%	90.01%	
Biswal et al. [53]	2018	EEG	RCNN	MGH SHHS	9000 subjects for training (MGH) 5224 subjects for training (SHHS) 1000 subjects for test (MGH) (SHHS) 580 subjects for test (SHHS)	Healthy—Mixed	9000 subjects for training; 1000 subjects for test 1000 subjects	5 stages	87.5% 77.7%		0.80 0.73
Cai et al. [54]	2021	EEG (1 channel)	Limited Penetrable Visibility Graph (LPVG) Graph-temporal fused dual-input CNN	Sleep-EDF	20 subjects	10 Healthy subjects 10 Subjects with mild sleep disturbance	Tenfold CV	2- 6 stages	91.97%		0.86
Chambonet al. [55]	2018	EEG EOG EMG	Spatial Filtering CNN	MASS	61 subjects	Healthy subjects	Fivefold CV Split: 41 Train -10 Val- 10 Test	5 stages	78.00%	72%	0.70
Chen et al. [56]	2020	EEG EOG EMG	Bi-LSTM-CNN	Sleep -EDF	39 subjects	Healthy subjects	Tenfold CV	5 stages	89.4%		
Cho et al. [57]	2022	EEG (6 channels) EOG (2 channels) EMG (1 ch)	StageNet (CNN + RNN)	SCHBC 2018–12-007	602 subjects	397 SDB (sleep apnea) subjects 205 Healthy subjects	482 subjects—training 48 subjects—validation 72 subjects—test	5 stages	90.4%		0.84
Choi et al. [58]	2020	Respiration, HR, BCG	LSTM	PSG recordings and PVDFdata (Seoul National University Hospital)	60 PSG	60 Healthy subjects	LOOCV 30 Training 10 Validation 20 Test	4 stages	73.9%		0.55
Delimayanti et al. [59]	2020	EEG (from single to multichannel)	High-Dimensional FFT Features SVM	Sleep-EDF	61 recordings for training (42 subjects)	61 Healthy subjects	Fivefold CV/tenfold CV	2- 6 stages	87.84% (avg)		
Dimitriadis Stavros et al. [60]	2018	EEG	Single-sensor ASSC Estimations of cross-frequency coupling with a multi-class Naïve Bayesian classifier	Sleep-EDF External Database	20 PSGs (Training) 77 PSG (Test)		LOOCV 20-fold CV—100 times	5 stages	94.40%		
Diykh et al. [61]	2020	EEG	Weighted undirected network. Least square support vector machine (LS-SVM)	Sleep-EDF ISRUC-Sleep	13 subjects 18 subjects	Mixed (healthy and SDB) subjects	Tenfold CV	6 stages	96.0% 96.74%		0.87 0.90
Dong et al. [62]	2018	EEG	Mixed Neural Network (MNN) Rectify Neural Network + LSTM	MASS	62 subjects	Healthy subjects	31-fold CV 30 subsets train 1 subset test	5 stages	85.92%	77.23%	
Dutt et al. [36]	2022	EEG (1 channel)	CNN CRFs (Conditional Random Fields)	Expanded Sleep-EDF	38 recordings (20 subjects)	Healthy subjects	20-fold CV	5 stages	86.8%		0.78
El Moaqet, Hisham et al. [63]	2022	EEG	TF images from wavelet transform GoogLeNet CNN, BiLSTM	DS-1 (Charité) DS-2(Umich)	20 subjects 61 subjects		20-fold CV	5 stages	91.2%		
Eldele et al. [39]	2021	EEG (1 channel)	Multi-resolution CNN Adaptive feature recalibration (AFR) Temporal context encoder (TCE) AttnSleep	Sleep-EDF-20 Sleep-EDF-78 SHHS	20 subjects 78 subjects 329 subjects	Healthy subjects	20-fold CV	5 stages	84.4% 81.3% 84.2%	78.1% 75.1% 75.3%	0.79 0.74 0.78
Eldele et al. [64]	2022	EEG	ADAST: Adversarial Domain Adaptation framework based on preserving attention mechanism and iterative Self-Training strategy	Sleep-EDF SHHS-1 SHHS2	20 subjects 42 subjects 44 subjects	Healthy subjects	60% training 20% validation 20% test	5 stages	74.0%	60.39%	
Erdenebayar et al. [65]	2020	EEG	SleepGRU	PSG recordings (Samsung Medical Center, Seoul, Korea)	89 subjects for training 23 subjects for testing	42 Healthy subjects/47 SDB (apnea) subjects 10 Healthy subject/12 SDB (apnea) subjects	89 Training 23 Test	3 stages	80.4%	80.7%	
Fan et al. [66]	2021	EOG	2-scale CNN RNN	MASS Sleep-EDF	62 PSG 39 PSG(20subjects)	Healthy subjects	LOOCV	5 stages	81.2% 76.3%	73.7% 69.3%	0.71 0.67
Feng et al. [67]	2021	EEGs	Time Attention Mechanism: Feature vector BiGRU	Sleep-EDF Self-Collected Dataset	8 subjects 83 recordings (21 subjects)	4 Healthy subjects/4 effects of temazepam—insomnio Healthy subjects	Fivefold CV	5 stages	92.18% 90.06%	91.77% 89.91%	0.87 0.86
Fonseca et al. [68]	2020	Heart rate variabilitiy (HRV)	LSTM	SOMNIA SIESTA Computer-Assisted Automated Scoring of Polysomnograms Using the Somnolyzer System	389 584 97	Mixed (healthy and SDB) subjects	Hold out 584 Training 1 (SIESTA) 97 Training 2 Validation 389 (SOMNIA)	4 stages (W/N1-N2/N3/REM)	75.9%		0.60
Fraiwan et al. [69]	2020	EEG (2 channels)	Bi-LSTM	Sleep-EDF	39 PSG recordings (20 subjects)	Healthy subjects	Tenfold CV	6 stages	97.28%	74.64%	0.72
Frilot et al. [70]	2018	EEG	CNN Fine-grained segments succeeding 30 s	ISRUC-Sleep	116 subjects		Tenfold CV	5 stages	92.00%		
Fu et al. [71]	2021	EEG (1 channel)	Attention Mechanism Bidirectional LSTM (AT-BiLSTM)	DRM-SUB PSEE Expanded Sleep-EDF (Test) DREAMS (Test)	20 subjects 20 subjects 153 subjects 20subjects	Healthy subjects	Fivefold CV	5 stages	83.78% 81.72%	82.14% 80.74%	0.77 0.75
Gaiduk et al. [72]	2018	Respiratory HR Movement	Multinomial Logistic Regression (MLR)	Charité Dataset	35 subjects	Healthy subjects	5 subjects for training 30 subjects for testing	3 stages 4 stages	72.00% 58.00%		0.67 0.50
Gaiduk et al. [73]	2022	Breathing, Movement	Multinomial Logistic Regression (MLR)	Charité Dataset	35 subjects	Healthy subjects	15 subjects for training 20 subjects for test	3 stages	73%		0.44
Ghasemzadeh et al. [74]	2019	EEG	D3TDWT Logistic smooth transition autoregressive (LSTAR) SVM	Sleep-EDF ISRUC-Sleep	13 subjects 10 subjects	Healthy subjects	Tenfold CV	5 stages (Sleep-EDF) 6 stages (Sleep-EDF) 5 stages (ISRUC-Sleep)	94.83% 93.92% 82.25%		0.89 0.87 0.77
Ghimatgar et al. [75]	2019	EEG	Random Forest Hidden Markov Model (HMM)	DRM-SUB ISRUC Sleep EDF Expanded Sleep-EDF	20 subjects + 100 subjects 8 subjects + 61 subjects	Healthy subjects	LOOCV p = 50%	5 stages 5 stages 5 stages/6 stages 5 stages/6 stages	79.08% 74.3% 87.59%/85.64% 80.55%/76.88%		0.73 0.71 0.81/0.78 0.73/0.68
Gopan et al. [76]	2020	EEG (5 channels)	Inter-channel covariance matrices Riemannian Manifold Ensamble with bagging	DREAMS	20 subjects	Healthy subjects	Tenfold CV		83.05%		0.79
Guillot et al. [77]	2020	EEG	SimpleSleepNet (Spectogram Signals and Frecuencies Reduction Gru with Attention Positional Embeddings Sequence Encoder)	DOD-H DOD-O	25 subjects 55 subjects	25 healthy subjects 55 SDB (OSA) subjects	DOD-H: LOOCV 18 Training 6 Validation 1Test DOD-O: tenfold CV 37 Training 12 Validation 6 Test		83.3% 89.0%	89.9% 88.3%	0.84 0.82
Guo et al. [78]	2022	Forehead electrophysiological (3 channels)	Light gradient boosting machine (LGB)	Expanded Sleep-EDF Own data	61 subjects 28 subjects	Healthy subjects	LOSO (leave one subject out)	5 stages	90.25%		0.85
Hasan, Md. et al. [79]	2020	EEG (Prefrontal,central,occipital)	PSD ResNet	DREAMS	20 subjects	Healthy subjects	Fourfold CV 75% Training and validation, 25 Test	5 stages	85.8%		
Hei et al. [80]	2022	EOG ECG	Feature Extraction Module Feature Fusion Module, XGBoost	SHHS2	69 subjects (PSG)	Healthy subjects	Fourfold CV	5 stages	83%		0.7
Hong et al. [31]	2021	EEG (1 channel)	SeqConfidNet Dropout Correct Rate (DCR)	SNUBH SHHS	702 2804	Mixed (healthy and SDB) subjects	70% Training 15% Validation 15% Test	5 stages	76% 82%	76% 82%	0.67 0.75
Horie et al. [37]	2022	EEGs, EOGs, EMG	Sleep-CAM: CNN	MASS + own data	109 subjects	Healthy subjects	Sevenfold CV	5 stages	86.9%		0.81
Huang et al. [40]	2022	EEG (1 channel)	CNN HMM	Sleep-EDFx	20 subjects	Healthy subjects	Tenfold CV and paired t-test	5 stages	84.6%		0.79
Huang et al. [81]	2020	EEG (2 channels)	ReliefF SVM	Expanded Sleep-EDF	30 subjects	Mixed (healthy and SDB) subjects	Tenfold CV 7:2:1	5 stages 6 stages	93.08% 92.34%		0.84
Huttunen et al. [82]	2022	Multichannel	Model 1(U-Net based) (PPG + SpO2) Model 2(U-Net based)(PPG + SpO2 + nasal pressure signal) Model 3 (U-Net based)(SpO2 + nasal pressure, EEG, oronasal thermocouple + RIP sum)	Princess Alexandra Hospital's Sleep Disorders Centre	877 subjects	Healthy subjects (suspected OSA)	80% Training 10% Validation 10% Test	5 stages	69% 70% 79%	68% 68% 79%	
Jadhav et al. [83]	2022	EEG (1 channel)	1D-CNN SWT-CNN STFT-CNN	Expanded Sleep-EDF (v1) Expanded Sleep-EDF(v2)	39 PSG recordings (20 subjects) 153 PSG recordings (78 subjects)	Healthy subjects	20-fold CV (Sleep-EDF-v1) tenfold CV (Sleep-EDF-v2)	5 stages	83.7%/82.4% 85.6%/82.5% 85.7%/82.7%		0.78/0.75 0.80/0.75 0.80/0.76
Jaoude et al. [84]	2020	EEG (4 channels)	CNN RNN	MGH-PSG scalpEEG	6431 subjects 112 subjects	Mixed (healthy and SDB) subjects 40 Healthy subjects/53 subjects with other pathologies (dementia..)	Fivefold CV	5 stages	81.15% 88.9%		0.74 0.78
Jia et al. [85]	2021	EEG EOG EMG	SleepPrintNet (a hierarchical recurrent neural network for the sequence-to-sequence classification problem)	MASS-SS3	62 subjects	Healthy subjects	31-fold CV	5 stages	88.8%	84.3%	0.83
Jiang et al. [87]	2019	EEG EOG EMG	Covariance Features Riemannian manifolds Bootstrap aggregation (Bagging) classifier	MASS	61 PSG	Healthy subjects	LOOCV	5 stages	81.20%	80.9%	0.72
Jiang et al. [86]	2019	EEG	Random Forest Hidden Markov Model (HMM)	MASS Sleep EDF Expanded Sleep EDF	89 subjects	Mixed (healthy and SDB) subjects	LOOCV	5 stages	80.8% 93.0% 92.6%	79.3% 93.0% 90.8%	0.71 0.86 0.86
Kang Dae et al. [88]	2018	EEG	Multitaper Spectral Estimation EEG Spectral Feature Straction Stage-Specific Kernel Density Estimation Hidden Markov Model	UC San Diego Sleep-EDF	65 (15 HN-50 OSA 15 (HN)	30 Healthy subjects 50 SDB (OSA) subjects	Fivefold CV	5 Stages			0.70
Karimzadeh et al. [89]	2018	EEG	Time/Frecuency Measure of Phase & Envelope Frequency Domain Segmentation Entropy Calculation (Shannon Entropy) Distributed decision tree classifier, KNN	Sleep -EDF SHHS	20 subjects 140 subject	Healthy subjects SDB subjects	LOO 60% validation/40% test 70% validation/30% test	5 stages	88.97% 83.17%		
Khalili et al. [90]	2021	EEG (1 channel)	CNN TCNN onditional Random Field layer Data augmentation	Sleep-EDF-2013 Sleep-EDF-2018	39 recordings 153 subjects	Mixed (healthy and SDB) subjects	LOOCV 39PSG SleepEDF-2013 tenfold CV 153 PSG SleepEDF-2018	5 stages	85.39% 82.46%		0.80 0.76
Khojandi A et al. [91]	2019	EEG (2 channels)	Random Forest	Sleep-EDF	20 subjects	Healthy subjects	20-fold CV	5 stages	74.00%		
Kim et al. [92]	2022	EEG (1 channel)	CNN Transformers	Own dataset (Hallym University College of Medicine (Chuncheon, Republic of Korea:No. 2021–03-005)	2274 subjects	Mixed (healthy and SDB) subjects	1590 subjects for training (70%) 341 subjects for validation (15%) 343 subjects for test (15%)	3 stages	91.4%	89%	0.84
Kim et al. [93]	2022	EEG EMG	CNN Feature-level fusion	Expanded Sleep-EDF	20 subjects	Healthy subjects	20-fold CV	5 stages	87.2%		
Kim et al. [94]	2022	HRV, Actigraphy	Random Forest	PSG recordings (Samsung Medical Center, Seoul, Korea)	30 subjects	Mixed (healthy and SDB) subjects	Paired t-test	5 stages	86.19%		0.97
Korkalainen et al. [95]	2020	PPG	CNN RNN	Clinical Dataset (Princess Alexandra Hospital (Brisbane, Australia) using Compumedics Grael acquisition system)	894 subjects	Mixed (healthy and SDB) subjects	Hold out 715 Training 90 Validation 89 Test	5 stages	64.1%		0.51
Korkalainen et al. [96]	2020	EEG EOG	CNN + LSTM	Expanded Sleep-EDF Clinical dataset	153 PSG 891 PSG	Healthy subjects Mixed (healthy and SDB) subjects	tenfold CV LOOCV 80% Training 10% Validation 10% Validation	5 stages	83.9% 83.8%		0.78 0.78
Kwon et al. [97]	2022	EEG (2 channels) EOG (2 channels)	CNN	ISRUC-Sleep Lab Dataset	100 subjects 4 subjects	Healthy subjects	60% training 20% validation 20% test	5 stages	88.85% 87.05%		0.85 0.83
Lee et al. [98]	2022	EEG (1 channel)	SleepExpertNet: Embedding vector generation (temporal spectra CNN) Temporal context modeling (multi-head attention + BiLSTM)	Sleep-EDFX-2018	153 subjects	Healthy subjects	Tenfold CV	5 stages	90.8%	86.7%	0.87
Li et al. [99]	2022	EEG	EEGSNet: CNNs + Bi-LSTM	Sleep-EDFX-8 Sleep-EDFX-20 Sleep-EDFX-78 SHHS	8 subjects 20 subjects 78 subjects 329 subjects	Healthy subjects	20-fold CV LOSO (leave-one-subject-out) 20-fold CV 20-fold CV	5 stages	94.17% 86.82% 83.02% 85.12%	87.78% 81.57% 77.26% 78.54%	0.91 0.82 0.77 0.79
Li et al. [100]	2022	EEGs	GCN (Graph neural network) CNN	Sleep-EDF ISRUC-SLEEP	20 subjects 10 subjects	Healthy subjects	20-fold CV tenfold CV	5 stages	91.0% 87.4%	89.0% 86.5%	0.88 0.84
Li et al. [101]	2021	PPG Actigraphy	CNN SVM	Emory Twin Study Follow-Up (ETSF)	105 subjects	No information given	tenfold CV 105 subjects ETSF	4 stages	68.62%		0.44
Li et al. [102]	2022	EEG(1 channel)	CAttSleepNet (Attention Module + CNN + BiLSTM)	Sleep-EDFX-2013 Sleep-EDFX-2018	39 recordings (20 subjects) 159 subjects (79 subjects	Healthy subjects	20-fold CV & tenfold CV + I53	5 stages	84.14% 80.81%	84.14% 80.81%	0.78 0.735
Li Xiaojin et al. [103]	2018	EEG	HyCLASSS: Feature extraction + Random Forest + Correction Rule	CCSHS CFS	116 subjects 82 subjects		Split:96 Train—102 Test	5 stages	85.95%	71.6%	0.8046
Li et al. [104]	2022	EEG ECG EOG EMG	MVF-SleepNet (VGG-16 + GRU…)	ISRUC-S3 ISRUC-S1	10 recordings 114 recordings	10 Healthy recordings 114 SDB recordings	Tenfold CV (ISRUC-S3) 90% Training—10% Test (ISRUC-S1)	5 stages	84.1% 82.1%	82.8% 80.2%	0.79 0.76
Ling et al. [105]	2022	EEG (1 channel)	RDB-DCGAN ( Residual Dense Block and DeepConvolutional Generative Adversarial Network) CNN	Expanded Sleep-EDF	40 subjects	Healthy subjects	70% Training 30% Test	5 stages	76.0%		
Liu et al. [106]	2021	EEG (1 channel)	Ensemble Empirical Mode Decomposition (EEMD) eXtreme Gradient Boosting (XGBoost) algorithm	Sleep-EDF DREAMS SHHS	16 recordings (8 subjects) 20 subjects 111 subjects	Healthy subjects	Fivefold CV	4/5 stages	93.1%/91.9% 86.4%/83.4% 87.5%/85.8%		0.87 0.77 0.79
Liu et al. [107]	2022	EEG	MAResnet (Multi-scale Attention Residual Net) BiGRU	Sleep-EDF UCD SHHS	20 subjects 20 PSG 5445 subjects	20 Healthy subjects 20 subjects with suspected SDB 5445 Mixed (healthy and SDB) subjects	Tenfold CV	5 stages	84.24% 79.34% 81.6%		0.78 (Sleep-EDF)
Liu et al. [108]	2020	EEG (1 channel)	Intrinsic geometry of sleep dynamics SVM	Expanded Sleep-EDF	83 recordings (42 subjects)	20 Healthy subjects 22 SDB subjects	LOOCV	5 stages	82.72%	75.91%	0.76
Liu et al. [109]	2022	EEG (single channel)	Deep neural network combining MSE(Multi-Scale Extraction) based U-structure and CBAM (Convolutional Block Attention Module)	Sleep-EDF-39 Sleep-EDF-153 SHHS	20 subjects/39PSGs 78 subjects/153PSGs 329 subjects/PSGs	Healthy Healthy With regular sleep	20-fold CV	5 stages	90.3% 89.7% 86.8%	86.2% 85.2% 83.5%	
Lu et al. [110]	2022	EEG (2 channels), EOGs (2 channels), EMG (1 channel)	CNN-RNN, weighted loss function (WLF)	Sleep-EDF-13 SHHS1	20 subjects/39 PSGs 5793 subjects/PSGs	Healthy Mixed	Nested CV (20-fold, tenfold) Split: 81%/9%/10%	5 stages	87.2% 89.1%	82.1% 81.4%	0.82 0.85
Luo et al. [111]	2022	Body movement ECG Abdominal breathing	Multi-head self-attention blocks coupled with CNN	MESA SHHS1	2237 subjects 5793 subjects/PSGs	Mixed Mixed	Fivefold CV	3 stages	82.9% 84.3%	79.2% 80.38%	0.70 0.70
Lv et al. [112]	2022	EEG (1 channel)	Multilevel temporal context network (MLTCN): CNN + TCN + HMM	Sleep-EDF-2013 Sleep-EDF-2018	20 subjects/39PSGs 7 subjects/153PSGs		20-fold CV tenfold CV	5 stages	84.2% 81.0%	77.0% 74.9%	0.78 0.74
Malafeevet al. [27]	2018	EEG (1 channel) EOG (2 channels) EMG (1 channel)	LSTM CNN-LSTM	Sleep laboratory, University of Zurich Sleep disorders center, Warsaw	18 subjects/54 PSGs 22 night PSG	Healthy Mixed	Split: 70%/15%/15% All dataset—test	5 stages			Healthy: ca. 0.8 for all except for N1 (< 0.5)
Matsumori et al. [113]	2022	Patch-type wearable EEG sensor (3 ch)	CNN-LSTM	Own dataset, PSG-1100 (NIHON KOHDEN)	25 subjects/26PSGs	Healthy, 6 suspected to have SDB at low level	Leave-one-out CV	5 stages	78.6%	73.4%	0.70
Mousavi et al. [114]	2019	EEG (1 channel)	CNN-BiRNN	Sleep-EDF-2013 Sleep-EDF-2018	61 PSGs 197 PSGs		20-fold CV tenfold CV	5 stages	84.3% 80.0%	79.7% 73.6%	0.79 0.73
Neng et al. [115]	2021	EEG (1 channel)	CCRRSleepNet: frame- level CNN and epoch-level RNN	Sleep-EDF-2013	20 subjects/39PSGs	Healthy	20-fold CV	5 stages	84.3%	79.8%	0.78
Olesen et al. [43]	2021	EEG (1 channel) EOGs (2 channels) EMG (1 channel)	Deep residual neural network	ISRUC MrOS SHHS WSC SSC	118 subjects/126PSGs 3932 PSGs 8444 PSGs 767 PSGs 2401 PSGs	Mixed	Split(ISRUC): 33%/33%/34% Split(other): 87.5%/2.5%/10%	5 stages	86.9%		0.80
Paisarnsrisomsuk et al. [116]	2021	EEG ( 2 channels) EOG (1 ch)	CNN	Sleep-EDF	20 subjects/39PSGs	Healthy	Nested 5 × 4 CV	5 stages	84.5%	78.1%	
Pan et al. [117]	2021	EEG + EOG + EMG + Resp + Temp	SVM	Sleep-EDF Hospital sleep data	20 subjects/38 PSG 31 subjects	Mixed Depressed/Healthy	Fourfold CV	4 stages	90.1% 78.6%/69.2%		0.87 0.71/0.58
Patanaik et al. [118]	2018	EEG (2 channels) EOG (2 channels)	dCNN	Own datasets (DS1-DS4) from Singapore and San Diego, USA	DS1: 120 subjects/1046 PSGs DS2: 52 subjects/284 PSGs DS3: 210 subjects/PSGs DS4: 77 subj/PSGs	Healthy Healthy Sleep disorders Parkinson´s disease	DS1, DS2—training DS3, DS4—test	5 stages	DS3: 81.4% DS4: 72.1%		DS3: 0.74 DS4: 0.60
Pathak et al. [38]	2021	EEG (2–8 channels) EOG (2–3 ch) EMG (1 ch)	CNN BiLSTM	SHHS1 MST	5793 PSGs 1418 PSGs	Mixed	Split: 81%/9%/10%	5 stages	85% 77%	79.0% 73.0%	
Pei et al. [119]	2022	EEG (1 channel) EOG (2 ch) EMG (1 ch)	CNN with GRU softmax classifier	UCDDB SHHS1	25 subjects/25 PSGs 700 PSGs		Split: 50%/20%/30%	5 stages	68.3% 83.2%		0.58 0.76
Perslev t al. [120]	2021	EEG (1 channel) EOG (1 channel)	U-Sleep, deep-learning-based system	16 independent clinical studies	15,660 subjects/19924 PSGs	Mixed	Split: training (at least 75%), validation (up to 10%, at most 50 subjects), testing (up to 15%, at most 100 subjects)	5 stages		79.0%	
Phan et al. [121]	2019	EEG + EOG/EEG + EOG + EMG	Multitask 1-max CNN	Sleep-EDF (expanded) MASS	20 subjects/39 PSGs 200 subjects	Suspected sleep disorder	Leave-one-subject-out CV 20-fold CV	5 stages	82.3% 83.6%	74.7% 77.9%	0.75 0.77
Phan et al. [122]	2019	EEG + EOG + EMG	SeqSleepNet: End-to-End Hierarchical RNN	MASS	200 subjects		20-fold CV	5 stages	87.1%	83.3%	0.82
Phan et al. [32]	2022	EEG (single channel)	SleepTransformer, a sequence-to-sequence sleep staging model	SHHS1 Sleep-EDF-78	5791 subjects 78 subjects/163 PSGs	Mixed	Split: 70%/30% tenfold CV	5 stages	87.7% 81.4%	80.1% 74.3%	0.83 0.74
Pini et al. [123]	2022	ECG	Deep-learning architecture: Neurobit-HRV	Physionet CinC Proprietary dataset	988 subjects/PSGs 52 subects/112 PSGs	Mixed	Previously trained on another dataset. Here all data is for test	3/4 stages	3: 82.1%/80.3% 4:73.0/70.3%		3:0.60/0.61 4:0.51/0.53
Qu et al. [124]	2020	EEG	ResNet-CNN	MASS Sleep-EDF	62 subjects/PSGs 20 subjects	31-fold CV 20-fold CV	5 stages	86.5% 84.3%	81.0% 79.0%		0.80 0.78
Qureshi et al. [125]	2019	EEG (1 channel)	CNN: GACNN SleepTuneNet	CAP Sleep Sleep-EDF	20 subjects 20 subjects		Tenfold CV	5 stages	95.6% 92.5%		0.94 0.90
Radha et al. [126]	2019	ECG HRV	LSTM	SIESTA	292 subjects/584 PSGs	Mixed	Fourfold CV	5 stages	77.0%		0.61
Seo et al. [127]	2020	EEG (1 channel)	CRNN: IITNet	Sleep-EDF-2013 MASS SHHS	20 subjects 62 subjects 5791 subjects	Mixed	20-fold CV 31-fold CV Split: 50%/20%/30%	5 stages	83.9% 86.5% 86.7%	77.6% 80.7% 79.8%	0.78 0.80 0.81
Sharma et al. [128]	2022	EEG (2 channels) EMG (1 ch) EOG (2 ch)	Wavelet-based Tsallis entropy features, EBT classifier	SHHS-1 SHHS-2	5793 subjects 2651 subjects	Mixed	10% holdout validation	3/5 stages	90.7%/84.3% 91.8%/86.3%		0.84/0.77 0.86/0.80
Shen et al. [129]	2020	EEG (1 channel)	Improved model based essence features (IMBEFs) Locality energy (LE) Dual state space models (DSSMs)	Dreams ISRUC	20 subjects/PSGs 10 subjects/PSGs		Tenfold CV	5 stages	79.9% 81.7%		0.76
Sokolovsky et al. [130]	2020	EEG (2 channels) EOG (1 channel)	CNN	Sleep-EDF (expanded)	20 subjects/39PSGs	Healthy	Fourfold CV	5 stages	81.0%	72%	
Sors et al. [131]	2018	EEG (1 channel)	CNN	SHHS-1	5793 subjects	Mixed	Split: 50%/20%/30%	5 stages	87.0%	78%	0.81
Sridhar et al. [132]	2020	Instantaneous heart rate (IHR), ECG	CNN	SHHS MESA CinC	561 subjects/800 PSGs 194 PSGs 993 subjects/PSGs	Mixed	Only test recordings are listed in the table	4 stages	77.0% 80.0% 72.0%		0.66 0.69 0.55
Sun et al. [133]	2019	EOG RR-intervals	CNN RNN	MASS (SS3) SA	62 PSGs 24 subjects/PSGs	Suspected sleep disorder	Leave-one-subject-out	5 stages/4 stages	84.4%/88.2% 74.3%/81.3%	78.1%/85.8% 74.0%/80.2%	0.77/0.80 0.66/0.71
Sun et al. [134]	2020	EEG EMG EOG	CNN + BLSTM	MASS (SS2-SS5)	147 subjects/PSGs	Suspected slep disorder	Leave-one-subject-out	5 stages	87.8%	81.8%	0.82
Sun et al. [135]	2021	EEG EOG	CNN	Sleep-EDF	20 subjects/39 PSGs	Healthy	20-fold CV	5 stages	83.4%	77.3%	0.77
Tao et al. [136]	2022	EEG (1 channel)	Cascaded CNN LSTM	Sleep-EDF-2013	20 subjects/39 PSGs	Healthy	Fivefold CV	5 stages	82.7%	76.8%	
Urtnasan et al. [137]	2022	Single-Lead ECG	Deep convolutional recurrent (DCR)	Proprietary dataset, Samsung Medical Center, Seoul, Korea	112 subjects	Control: 52 subjects, apnoea: 60 subjects	Training set: 89 subjects, test set: 23 subjects	5 stages 3 stages	74.2% 86.4%		
Vallat et al. [138]	2021	EEG (1 channel) EOG (1 channel) EMG (1 ch)	LightGBM	MESA, CFS, CCSHS, SHHS, MrOS, CHAT, HomePAP, DOD (healthy and obstructive)	3798 PSGs	Mixed	Training: 3163 PSGs Test: 585 PSGs/50 PSGs (DOD-obstructive)	5 stages	87.5%/84.3%	/74.0%	0.82/
van der Plas et al. [33]	2021	EEG EMG EOG ECG SaO2 Position Snore sound	Emission models and the random forest	1: Dataset from 10 medical centers in Belgium, the Netherlands and Austria 2: Proprietary dataset from a single center	1: 5884 PSGs 2: 50 PSGs	1: each PSG is scored by one expert 2: scored by four, five, six experts or seven	1: Split: 33.3%/33.3%/33.3% 2: Test only	5 stages	1: 80.5% 2: 82.7%		1: 0.73
Vanbuis et al. [34]	2020	EEG EMG EOG	Random forest, interpretable	Proprietary dataset, Institut de Recherche en Santé Respiratoire	400 PSGs	Suspected having OSA subjects	Split: 75%/25%	5 stages	77.8%		0.69
Vanbuis et al. [139]	2022	Pulse oximetry Respiration flow and effort Actigraphy Ambient light Tracheal sound	Multilayer perceptron classifier	Proprietary dataset, Institut de Recherche en Santé Respiratoire	400 PSGs	Suspected having OSA subjects	Split: 75%/25%	5 stages 3 stages	62.4% 78.5%		0.48 0.60
Wang et al. [140]	2022	EEG	Deepsleepnet network (CNN, bi-LSTM), LightGBM	MASS Sleep-EDF (expanded)	200 subjects 20 subjects	Suspected sleep disorder	Training: MASS, Sleep-EDF Test: Sleep-EDF Leave-one-out CV	5 stages	87.8%		
Wang et al. [141]	2022	EEG (1 channel)	Multiscale dual attention network (MSDAN)	Sleep-EDF Sleep-EDFx	8 PSGs 197 PSGs		20-fold CV	5 stages	91.7% 90.4%	82.3% 79.5%	0.87 0.83
Wei et al. [142]	2019	Single-Lead ECG	LSTM	Proprietary dataset, Xijing Hospital, Fourth Military Medical University	373 subjects/PSGs	depression or schizophrenia	Split: 238/60/75 recordings	3 stages 4 stages 5 stages	84.1% 77.8% 71.2%		0.58 0.55 0.52
Wongsirichot et al. [143]	2019	Multichannel	k-Nearest Neighbors (the best results)	SHHS	2535 PSGs	Mixed	Tenfold CV	5 stages	83.8%	69.4%	
Yan et al. [144]	2021	EOG, EEG, EMG, ECG	2d CNN LSTM	SHHS-1 ISRUC Sleep-EDF	100 PSGs 99 PSGs 19 PSGs	RDI3P < 5 mixed health state 2nd night	Fivefold CV	5 stages	87.0% 86.0% 86.0%		0.81 0.82 0.81
Yang et al. [41]	2022	EEG	Conditional random fields (CRF), CNN-LSTM	Sleep-EDF-20 DRM-SUB SVUH-UCD	20 subjects/39 PSGs 20 subjects/PSGs 25 subjects	Healthy Healthy With sleep apnoea	20-fold CV 20-fold CV 25-fold CV	5 stages	85.2% 83.2% 74.8%	78.1% 76.9% 69.9%	0.79 0.77 0.66
You et al. [145]	2022	EEG (1 channel)	Bi-LSTM, Fractional Fourier transform (FRFT)	Sleep-EDF MASS	20 subjects 62 subjects	Healthy subjects	20-fold CV	5 stages	81.6% 84.3%	74.7% 77.8%	0.75 0.77
Yu et al. [146]	2022	EEG (1 channel)	Deep neural network—MRASleepNet: a feature extraction (FE) module, an MRA module, and a gMLP module	Sleep-EDF-20 Sleep-EDF-78 CAP	20 subjects 78 subjects 63 subjects	Healthy and mixed subjects	20-fold CV tenfold CV Split: 70%/10%/20%	5 stages	84.5% 81.4% 74.3%	78.9% 75.4% 65.6%	0.79 0.74 0.65
Yulita et al. [147]	2018	EEG EMG EOG	The fast convolutional method	Physionet Proprietary dataset: Mitra Keluarga Kemayoran Hospital	25 subjects 10 subjects		Leave-one-out CV	5 stages		73.5% 56.3%	
Zhang et al. [148]	2022	EEG (dual channel)	Random forest, rule-set creation based on data-mining, Ontology-Based Modeling (OBM)	Sleep-EDF (expanded)	42 subjects/61 PSGs	Healthy and with mild difficuty in falling asleep	Split: 50%/50%	5 stages	89.1%	85.9%	0.81
Zhang et al. [149]	2022	EEG	Co-attention meta sleep staging network (CMS2-net), semi-supervised	Proprietary: Peking University Sixth Hospital DOD-H DOD-O	48 subjects 25 subjects 55 subjects	Mixed (5 subsets) Healthy OSA patients	Split: 60%/20%/20%	5 stages	74%-81% 80% 78%	53%-69% 69% 68%	0.62–0.73 0.71 0.68
Zhang et al. [150]	2021	EEG (1 channel)	Competition convolutional neural network (C-CNN)	UCD Sleep-EDF(expanded)	25 subjects 42 subjects	With sleep-respiration disorder	Leave-one-out CV 20-fold CV	5 stages	77.2% 83.5%	84.0%	0.73
Zhang et al. [44]	2019	EEG EMG EOG	Deep neural network combined recurrent and convolutional structures	SHHS-1 MrOS SOF	5793 subjects 2907 subjects 461 subjects	Mixed	Training (SHHS): 5213 subjects Test (SHHS): 580 subjects, MrOS, SOF	5 stages	87.0%	87.0% 79.0% 77.0%	0.82 0.70 0.68
Zhang et al. [151]	2020	EEG (2 channels) EOG (2 channels) EMG (1 channel)	CNN	Proprietary dataset: Beijing Tongren Hospital Sleep-EDF-2013	294 PSGs 20 subjects	Snoring	Split: 122/20/152 Sleep-EDF—test	5 stages	81.8% 83.6%	81.5% 78.1%	0.73 0.77
Zhang et al. [152]	2018	Heart rate (from PPG), Actigraphy	Multi-level feature learning RNN (BiLSTM)	Proprietary dataset: General Hospital of the Air Force, PLA, Beijing, China	39 subjects	Healthy	Leave-one-out CV	5 stages		60.5%	
Zhong et al. [153]	2021	EEG (1 channel)	Multiscale residual convolutional neural network (MRCNN)	Sleep-EDF(expanded) CinC2018	197 PSGs 500 PSGs	Mixed	Fivefold CV Subject CV, Split 400/100	5 stages	92.1% 89.2%		0.74 0.62
Zhou et al. [154]	2022	EEG (1 channel)	SingleChannelNet (deep neural network)	CCSHS (children) Sleep-EDF (expanded)	515 subjects 78 subjects/163PSGs		Fivefold CV	5 stages	90.2% 86.1%		0.87 0.81
Zhou et al. [155]	2020	EEG (1 channel)	Random forest (RF) and LightGBM (LGB)	Sleep-EDF Sleep-EDF (expanded)	8 subjects/PSGs 153 PSGs		Twofold CV (subject-independent)	5 stages	82.4% 85.3%	75.1% 83.4%	0.72 0.69
Zhu et al. [42]	2020	EEG (2 channels) EOG (1 channel) EMG (1 channel)	Multi-branch convolutional neural network Residual attention method	Sleep-EDFx UCDDB	20 subjects/39 PSGs 25 subjects	Healthy Sleep disordered breathing	Leave-one-subject-out	5 stages	85.8% 79.4%	81.2% 78.8%	0.80 0.73
